# Efficient Nonviral Transfection of Human Bone Marrow Mesenchymal Stromal Cells Shown Using Placental Growth Factor Overexpression

**DOI:** 10.1155/2018/1310904

**Published:** 2018-12-24

**Authors:** Winston Y. Cheung, Owen Hovey, Jonathan M. Gobin, Gauri Muradia, Jelica Mehic, Carole Westwood, Jessie R. Lavoie

**Affiliations:** Centre for Biologics Evaluation, Biologics and Genetic Therapies Directorate, Health Products and Food Branch, Health Canada, Ottawa, Ontario, Canada

## Abstract

**Background:**

Human mesenchymal stromal/stem cells (hMSCs) hold great therapeutic potential due to their immunomodulatory and tissue regenerative properties. Enhancement of biological features of hMSCs by transfection has become a focus of investigation for cell- and gene-based therapies. However, many of the current transient transfection methods result in either low transfection efficiency or high cytotoxicity.

**Methods:**

In order to find a transfection method that would address the current issues of low transfection efficiency and high cytotoxicity, 6 commercially available cationic lipid and polymer reagents were tested on human bone marrow-derived MSCs (hBM-MSCs) using GFP as a reporter gene. One transfection method using TransIT-2020 was selected and tested with an emphasis on cell quality (viability, identity, and yield), as well as efficacy with a human placental growth factor (PlGF) plasmid.

**Results:**

TransIT-2020 yielded the highest fluorescence signal per cell out of the methods that did not decrease cell recovery. Transfecting GFP to 5 hBM-MSC donors using TransIT-2020 yielded 24–36% GFP-expressing cells with a viability of 85–96%. hBM-MSC identity was unaffected as CD90, CD105, and CD73 markers were retained (>95%+) after transfection. When this method was applied to PlGF expression, there was up to a 220-fold increase in secretion. Both growth and secretion of PlGF in overexpressing hBM-MSC were sustained over 7 days, confirming the sustainability and applicability of the TransIT-2020 transfection system.

**Discussion:**

We report a simple and efficient method for transient transfection that has not been reported for hBM-MSCs, encompassing high levels of plasmid expression without significant changes to fundamental hBM-MSC characteristics.

## 1. Introduction

Multipotent human mesenchymal stromal/stem cells (MSCs) are a heterogeneous population of stromal cells capable of supporting hematopoiesis, mediating tissue repair and immunomodulation [1]. Based on these essential biological functions, their proliferative capacity and their immunoprivileged trait, MSCs have become a major focus of investigation for many potential therapeutic applications. This includes cardiovascular, immunological, and neurodegenerative diseases with unmet clinical needs [2]. Although MSCs can be obtained from many tissue sources, the most common are those derived from bone marrow (hBM-MSCs) [3]. Notably, the clinical utility of hBM-MSC treatments could be greatly enhanced by genetically modifying certain biological features aimed at improving important traits such as survival and potency [4, 5]. Strategies range from using cells as a vector for the delivery of therapeutic agents aimed at tissue repair/regeneration [6, 7] to delivering antitumour agents toward malignant sites in cancer [8–10]. When genetically modifying hBM-MSCs, it is important not to compromise cell quality (viability, identity, and yield), potency and safety since all are important aspects to consider when translating research findings to the clinic [11, 12].

Genetic modification of mammalian cells through exogenous nucleic acids can be performed through viral (retro-, lenti-, or adenoviruses) and nonviral methods, such as cationic lipids and polymers. Due to the difficulty of transfecting hBM-MSCs, the high efficiency of some viral gene delivery systems has made them an attractive method of transfection [4, 13]. Viral transfections, however, are associated with significant health risks, as they can elicit immunogenic responses and uncontrolled transgene expression, which can lead to changes in the characteristics of genetically modified cells [14–17].

While nonviral methods such as cationic lipids and polymers do not typically have transfection efficiencies as high as viral methods, they are less labour intensive and have lower immunogenicity [18–23]. These cationic lipids and polymers create complexes through electrostatic interactions with the phosphate backbone of the nucleic acids. Both cationic lipid and polymer transfection conform to the scheme of masking the DNA's negative charge through the use of positively charged lipid or synthetic polymer chains, respectively, in order to promote endosome uptake for gene delivery which is subsequently introduced in the cell via endocytosis [24, 25]. In addition, cationic lipids and polymers can deliver larger transgenes and can be just as effective as viral methods when used to treat noninherited diseases [26–29]. As of 2012, lipofection is the second most popular nonviral gene modification system in clinical trials with a usage of 5.9% [30]. This study is aimed at finding an effective means of transient transfection that maintains high cell quality by comparing both reported and unreported transfection systems that are commercially available for hBM-MSCs.

## 2. Material and Methods

### 2.1. hBM-MSC Cell Culture

hBM-MSC cell lines purchased for this study (hBM-MSC #15, #12RB, #37RB, #48RB, #56RB, and #85RB) were derived from the bone marrow of 6 healthy male donors by the companies under “informed consent” (Supplementary Table 1). hBM-MSC #15 was derived from mononuclear cells (cat.#1M-125D) isolated by Lonza (Lonza, Walkersville, MD, USA) and grown using serum-free MesenCult-XF medium (cat.#05429, STEMCELL Technologies; Vancouver, Canada) in flasks coated with MesenCult-SF Attachment Substrate (cat.#05424, STEMCELL Technologies). All hBM-MSC RB (cat.#MSC-001) cells were generated from a master cell bank and characterized by RoosterBio Inc. (Frederick, MD, USA). RB cells were grown in low-serum-containing RB complete medium composed of hBM-MSC High Performance basal medium and hBM-MSC Media Booster GTX supplement (cat.#KT-001, RoosterBio Inc.). We further performed hBM-MSC characterization using population doublings, hBM-MSC surface markers, and differentiation potential (Supplementary Table 1). hBM-MSCs were differentiated following a previously described protocol [31]. For RB cells, StemXVivo Chondrogenic Base Media (cat.#CCM005, R&D Systems; Minneapolis, MN, USA) were used for the basal media for chondrocytes. Minimum Essential Medium *α* (Thermo Fisher Scientific) with HyClone Defined FBS (Thermo Fisher Scientific) was used as basal media for adipocytes and osteocytes. Antibodies were obtained from eBioscience (San Diego, CA, USA) and BD Biosciences (Mississauga, Canada); details can be found in Supplementary Table 2. All flow cytometry samples were analysed using LSRII flow cytometer (BD Biosciences) and FlowJo V10 (FlowJo LLC, Ashland, OR, USA). A minimum of 40,000 events were recorded.

### 2.2. Plasmid Preparation

pCMV6-AC-GFP (cat.#PS100010) and pCMV6-AC-PlGF (cat.#SC320206) plasmids were purchased from OriGene (Rockville, MD, USA). pCMV6-AC-GFP was amplified using Alpha-Gold competent cells (cat.#CC100001, OriGene) and the pCMV6-AC empty vector was generated from glycerol stocks (cat.#PS200020-GLY, OriGene). These plasmids were purified using an EndoFree Plasmid Maxi Kit (cat.#12362, Qiagen; Toronto, Canada). The concentration and purity (260/280 > 1.80 and 260/230 > 2.0) were assessed before each use by NanoDrop 2000 (Thermo Fisher Scientific, Waltham, MA, USA).

### 2.3. Screen of Cationic Lipids and Polymers for Transfection in a Multivariate Analysis

**Figure 1:**
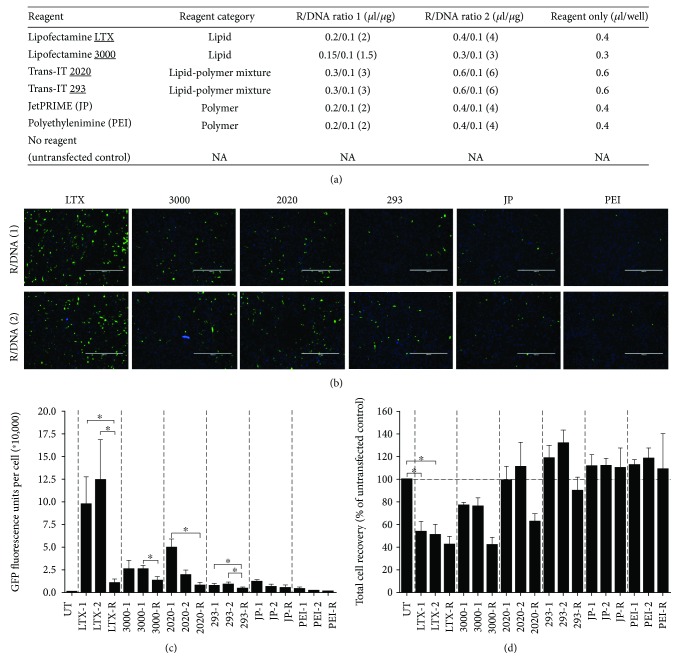
A multivariate approach to test the transfection efficiency of 6 commercially available cationic lipids and polymers using hBM-MSC #15. (a) Table of conditions for the 96-well multivariate transfection screening experiment with an untransfected control (UT) where no transfection reagent or DNA is added. (b) Representative 4X fluorescent overlay images of GFP+ cells (green) and Hoechst nuclei staining (blue) for the 6 commercially available cationic lipids and polymers assayed on hBM-MSCs. Images were taken after 24 hours at 2 different reagent/DNA (R/DNA) ratios per condition (1 and 2). Scale bars represent 1000 *μ*m. (c) GFP fluorescence was quantified using a plate reader at 24 hours using 2 different R/DNA ratios for all 6 transfection conditions, along with their respective transfection agent control. (d) Total cell recovery quantification of all transfection conditions assayed on hBM-MSCs after 24 hours at 2 different R/DNA ratios and their respective transfection agent control. Cell recovery is expressed as a percentage of total cells measured in an experimental condition divided by total cells measured in the untransfected control. Results are from 4 independent experiments using 3 technical replicates with bars representing means ± S.E.M. Statistical significance was obtained using a one-tailed *t*-test for (c) and a one-way ANOVA with a Dunnet post hoc analysis for (d). ^∗^ *p* < 0.05 and ^∗∗^ *p* < 0.01.

The multivariate 96-well plate method published by Sandbichler et al. [32] was adapted to evaluate the transfection efficiencies of 6 commercially available cationic lipids and polymer reagents; Lipofectamine LTX (Invitrogen) cat.#15338-100, Lipofectamine 3000 (Invitrogen) cat.#L3000-008, Trans-IT 2020 (Mirus) cat.#MIR 5400, Trans-IT 293 (Mirus) cat.#MIR 2700, jetPRIME (Polyplus) cat.#114-01, and polyethylenimine (Sigma) cat.#408,727. hBM-MSC #15 cells were seeded for transfection (6.0 × 10^3^ cells/well, 96-well plate), and medium was replaced after 24 hours. Transfection with the GFP plasmid (pCMV6-AC-GFP) was performed according to each manufacturer's protocol under different conditions outlined in Figure 1(a). hBM-MSCs were incubated with their respective complexes for 24 hours before being incubated in Live Cell Imaging Solution (cat.#A14291DJ, Invitrogen; Carlsbad, CA, USA) with 1 : 10000 Hoechst 33342 (Invitrogen) for 1 hour prior to quantification of total GFP signal and cell recovery by scanning plate at (GFP ex:485/20, em:528/20 and Hoescht ex:360/40, em:460/40) with the Synergy 2 (Thermo Fisher Scientific) plate reader. Total cell recovery was generated by interpolation from a seeding density standard curve (0–15,000 cells per well) using Hoechst 33342. This was also used to normalize GFP expression per cell by expressing the ratio of total GFP fluorescent signal (in RFU) over the number of cells interpolated in each well.

### 2.4. Quantitative Assessment of Transfection Efficiency and Cell Quality in a 6-Well Plate

24 hours after seeding, hBM-MSC #15 (1.5 × 10^5^ cells/well of a 6-well plate) culture medium was replenished. Cells were then incubated with the TransIT-2020: plasmid complex, with a reagent/DNA (R/DNA) ratio of either 2.0 (5 *μ*l of reagent to 2.5 *μ*g of DNA), 3.0 (7.5 *μ*l reagent to 2.5 *μ*g of DNA), or 4.0 (10.0 *μ*l reagent to 2.5 *μ*g of DNA) for 24 hours before harvesting to stain for cell surface markers.

For hBM-MSC #37RB, cells were seeded at 1.0 × 10^5^ cells/well 24 hours before transfection to achieve 70–90% confluence in a 6-well plate. Cells were then incubated with the TransIT-2020: plasmid complex at a ratio of 3.0 in complete medium for 24 hours (24h-CM), in Opti-MEM (cat.#51985034, Thermo Fisher Scientific) for 4 hours before being replaced with CM for the remaining 20 hours (4h-OM) or in Opti-MEM for a total of 24 hours (24h-OM) (Supplementary Figure 2). For flow cytometric analyses, cells were washed and suspended in PBS containing 2% FBS and 2 mM EDTA then incubated with 4 nM SYTOX Orange (cat.#S34862, Thermo Fisher Scientific) for 5 minutes to assess cell viability. Gating was done to select for the cell population, dismiss doublets, and exclude dead cells using the SYTOX Orange dye.

Transfection with the 4h-OM condition was repeated with 4 additional hBM-MSC donors (#12RB, #48RB, #56RB, and #85RB) while still including donor #37RB. Cells were tested for CD90, CD73, and CD105 surface marker expression (Supplementary Table 2) and ability to differentiate after transfection. Cytotoxicity was also evaluated posttransfection using the Pierce™ Lactate Dehydrogenase (LDH) Cytotoxicity Assay Kit (cat.#88954, Thermo Fisher Scientific), following the manufacture's protocol on the cell-conditioned media.

### 2.5. Assessment of Transfection Efficiency by ELISA

hBM-MSC #37RB cells were transfected using a R/DNA ratio of 3.0 in the 4h-OM transfection condition with a placental growth factor (PlGF) plasmid (pCMV6-AC-PlGF), an empty vector control (pCMV6-AC), or transfection reagent only control (R). After 24 hours, cell growth was evaluated and cell-conditioned media were collected and frozen at −80°C for later quantification of human PlGF levels with a PlGF ELISA kit (cat.#ab100629, Abcam; Cambridge, UK) according to the manufacturer's protocol. The transfection procedure was scaled up to 750,000 cells in a T75 flask where PlGF abundance levels and cell growth were also evaluated from PlGF-overexpressing hBM-MSCs. After transfection, hBM-MSC #37RB cells were seeded at 37,500 cells/well in a 6-well plate to assess growth and PlGF levels after 1, 5, and 7 days. The percent cell growth was calculated by dividing the live cell count at each time point by the initial cell seeding and multiplying by 100. Live cells were determined using Trypan blue exclusion with a % CV of less than 20%.

### 2.6. Microscopic Assessment

Representative pictures of hBM-MSC #15 were taken 24 hours after transfection using the EVOS FL microscope (Invitrogen) (Objective 4X, Camera Sony ICX285AQ color CCD, and EVOS® FL Cell Imaging System Software). Representative pictures of hBM-MSC RB cells were taken using an Axio Observer Z1 microscope (Carl Zeiss, Oberkochen, Germany), with 10-20X objectives, Axiocam 506 camera, A-Plan10x/0.25 (or 20x/0.3) Ph1 objective lenses, and ZEN 2 Pro acquisition software.

### 2.7. Statistical Analyses

**Figure 2:**
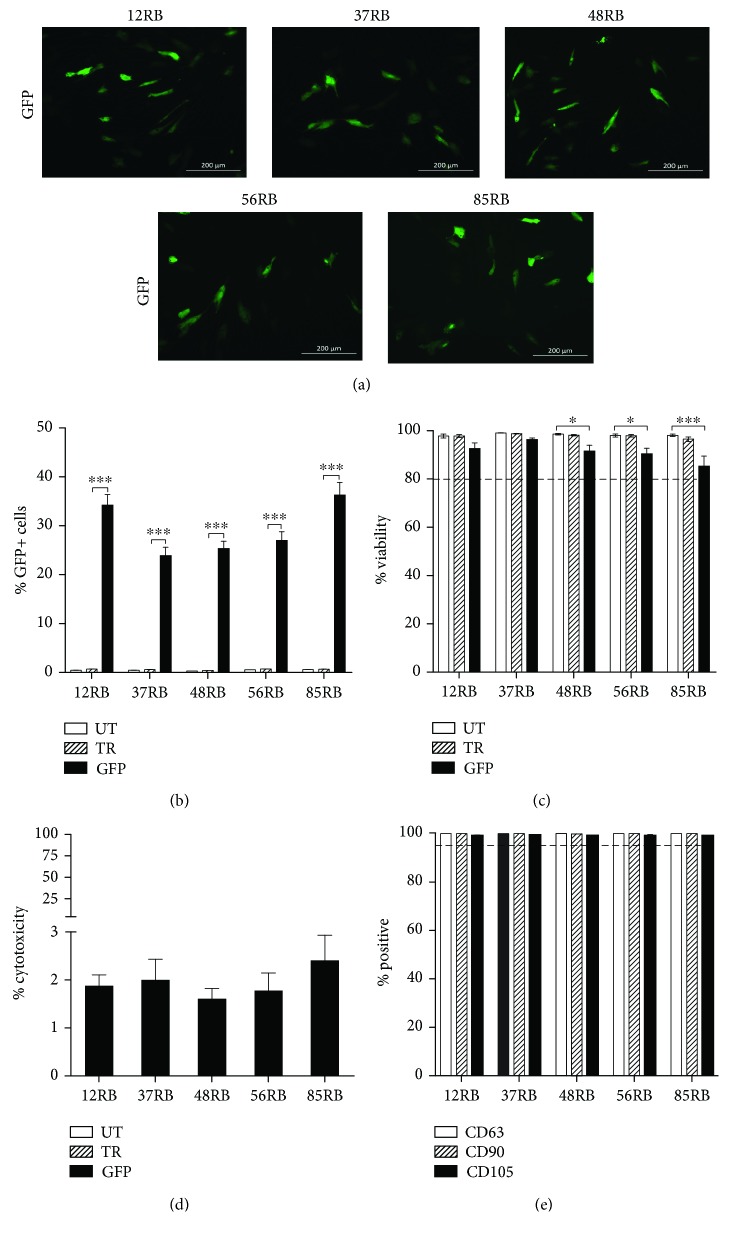
Effect of donor variability on hBM-MSC cytotoxicity, viability, surface marker profiling, and efficiency after transfection. (a) Representative 20X fluorescent images of GFP-transfected cells using TransIT-2020 where green represents GFP+ cells. Transfection was done 24 hours prior on 5 different hBM-MSC cultures (12RB, 37RB, 48RB, 56RB, and 85RB). Scale bars represent 200 *μ*m. (b) Quantification by flow cytometry of percent GFP+ cells of 5 hBM-MSC donors. (c) Percentage of viable cells quantified by flow cytometry using SYTOX Orange. (d) Percentage of cytotoxicity in the cells following transfection via quantification of lactate dehydrogenase (LDH). (e) Percentage of cells positive for hBM-MSC CD73, CD90, and CD105 surface markers after transfection. Results from 4 (a, b, c, and e) or 3 (d) independent experiments with technical duplicates, where error bars represent S.E.M. Statistical significance was obtained using multiple *t*-tests followed by a Holm-Sidak correction. ^∗^ *p* < 0.05, ^∗∗^ *p* < 0.01, and ^∗∗∗^ *p* < 0.001.

**Figure 3:**
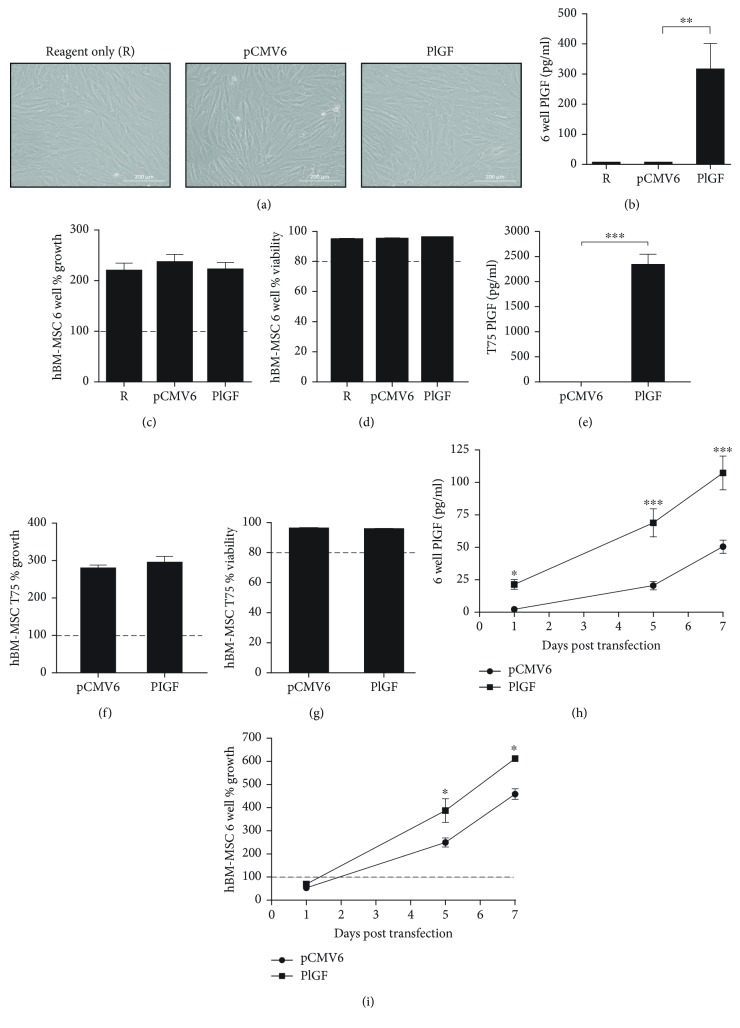
Sustained hBM-MSC growth and secretion of placental growth factor after TransIT-2020 transfection. (a) Representative 20X phase contrast images showing cell morphology of hBM-MSCs with reagent only control (R) and the 4h-OM condition with either the pCMV6 empty control or pCMV6-PlGF vector. Scale bars represent 200 *μ*m. (b) Quantification of PlGF secretion per well in a 6-well plate (pg/ml) in the hBM-MSC cell-conditioned media 24 hours after transfection by ELISA. (c) Amount of viable cells present 24 hours after transfection expressed as a percentage of initial seeding density in a 6-well plate. (d) Percentage of viable cells after transfection in a 6-well plate. (e) Quantification of PlGF secretion (pg/ml) per T75 flask. (f) Amount of viable cells present 24 hours after transfection in a T75. (g) Percentage of viable cells after transfection in a T75. (h) Secretion of PlGF (pg/ml) throughout the 7 days after transfection. (i) Amount of cell growth up to 7 days after transfection, expressed as a percentage of viable cells measured divided by the initial seeding of 37,500 cells. Results from 3 (a–d) or 4 (e–i) independent experiments with technical duplicates. Error bars represent S.E.M. Statistical significance was obtained using a one-tailed *t*-test for (b) and a two-way ANOVA with a Bonferroni post hoc analysis for (f–g). ^∗^ *p* < 0.05 and ^∗∗^ *p* < 0.01.

For each experiment, data is presented as the average with the standard error of the mean (S.E.M.) of at least 3 independent experiments (Figure 1, *n* = 4; Figure 2, *n* = 3 − 4; and Figure 3, *n* = 3 − 4 experiments), with technical duplicates or triplicates as indicated in figure legends. Significance was analysed by a one-way or two-way analysis of variance (ANOVA) with a Dunnet or Bonferroni post hoc analysis or, when appropriate, a one- or two-tailed student *t*-test, corrected using the Holm-Sidak correction when required. Statistical analyses were performed using GraphPad Prism version 6.0 (GraphPad Software Inc., La Jolla, CA, USA). A *p*-value of <0.05 was considered statistically significant, and significance differences are marked with a single asterisk (*p* < 0.05), double asterisks (*p* < 0.01), or triplicate asterisks (*p* < 0.001).

## 3. Results

### 3.1. Selection of TransIT-2020 out of 6 Cationic and Lipid Polymers for hBM-MSC Transfection

Out of the 6 commercially available cationic lipids and polymers tested on hBM-MSC#15 in a 96-well plate using 2 transfecting conditions (Figure 1(a)), 4 reagents (Lipofectamine LTX, Lipofectamine 3000, TransIT-2020, and TransIT-293) showed a significant increase in GFP expression per cell compared to their reagent only controls (R), as reported in relative fluorescence units (RFUs) (Figures 1(b) and 1(c)). All transfection reagents, except Lipofectamine LTX, showed no significant change in cell recovery (Figure 1(d)), an important aspect when genetically modifying cells as final cell yield is often important for clinical translation. Since the TransIT-2020 transfection system showed the highest GFP expression without decreasing cell recovery, it was selected for further validation of its performance.

### 3.2. Successful Scale up Using TransIT-2020 while Maintaining GFP Expression, hBM-MSC Surface Marker Profile, and Viability

Focusing our investigation on the TransIT-2020 system, we scaled up to a 6-well plate to assess the feasibility of the method at a larger scale and tested 3 different R/DNA ratios (2.0, 3.0, and 4.0 R/DNA), since R/DNA ratios can affect transfection efficiency. The R/DNA ratio of 3.0 had the highest percentage of GFP+ cells (35.4 ± 6.6%) and mean fluorescence intensity (MFI) (39,390 ± 6,340 MFI), without significantly affecting surface marker expression (>95%) while maintaining over 80% viability (Supplementary Figure 1), which led us to select a R/DNA ratio of 3.0 and pursue further testing.

TransIT-2020 was further tested through an additional hBM-MSC donor, hBM-MSC #37RB to verify applicability to hBM-MSC generated under different culture conditions. In addition to the 24-hour incubation period in complete medium (24 h-CM) recommended by the manufacturer, we examined 2 conditions using Opti-MEM medium with a 4-hour (4h-OM) or 24-hour (24h-OM) incubation time of the R/DNA complexes to verify whether efficiency would be increased under serum-free conditions (Supplementary Figure 2).

The highest percentage of GFP+ cells was obtained in the 24h-OM condition (52.1 ± 6.4%), whereas the 4h-OM and 24h-CM conditions led to 32.0 ± 4.8% and 38.7 ± 7.8% GFP+ cells, respectively (Supplementary Figure 3(b)). All conditions were significantly expressing more GFP than R (0.53 ± 0.09%, 0.37 ± 0.05%, and 0.35 ± 0.07%, respectively). However, the 4h-OM condition achieved the highest mean fluorescence intensity (MFI), a measure of the amount of GFP expressed per cell, with 39,469 ± 5185 MFI compared to the 24h-OM (27,500 ± 3,555 MFI) and the 24h-CM conditions (21,178 ± 3661 MFI) (Supplementary Figure 3(c)). Although the 24h-OM condition had the highest percentage of GFP+ cells (Supplementary Figure 3(b)), the uniform GFP expression (Supplementary Figure 3(a)) and high MFI (Supplementary Figure 3(c)) of the 4h-OM indicated that this condition led to genetically modified hBM-MSCs with the most effective transgene expression overall. This prompted us to proceed with the 4h-OM condition for future experiments.

The 4h-OM condition was therefore tested with 4 additional hBM-MSC RB donors in order to determine the amount of donor-donor variability as well as the applicability to other hBM-MSC donors. A GFP expression range of 24–36% (Figure 2(b)), viability of 85–96% (Figure 2(c)), and LDH cytotoxicity of 1.7–2.3% (Figure 2(d)) were obtained, indicating effective GFP expression without affecting cell health. After transfection, CD73, CD90, and CD105 surface marker expression levels were >95%+ (Figure 2(e)) and all cells tested were able to differentiate into osteocytes, adipocytes, and chondrocytes (Supplementary Table 3), confirming that hBM-MSC identity was not changed.

### 3.3. Application of 4h-OM Transfection Condition to PlGF Overexpression hBM-MSCs

The 4h-OM transfection condition based off GFP reporter plasmid optimisation workflow was then tested for applicability using a plasmid encoding for PlGF. In addition, since hBM-MSC 37RB cells had the least expression of the GFP reporter plasmid among all donors tested, it was selected to prove the applicability of the optimised 4h-OM condition using the PlGF plasmid. We found significantly elevated levels of secreted PlGF in PlGF-transfected hBM-MSCs (315 ± 50 pg/ml) compared to those of the cells transfected with an empty plasmid (5.25 ± 0.75 pg/ml), without significantly affecting cell growth (268 ± 14%) or viability (96.1 ± 0.24%), indicating the efficient production of PlGF without comprising cell health (Figures 3(b)–3(d)). To show the method's potential to be further scaled up to produce clinically relevant doses of genetically modified hBM-MSCs, the same workflow was scaled up to a T75, yielding a 220-fold increase in PlGF secretion levels with 2,353 ± 195 pg/ml, which is significantly higher compared to the empty plasmid transfected cells with 10.2 ± 1.4 pg/ml. PlGF-overexpressing cells maintained 293 ± 17.9% growth and 95.9 ± 0.8% viability (Figures 3(e)–3(g)). To know whether the cells can sustain increased PlGF production over time, a potentially important characteristic for clinical use, the overexpressing hBM-MSCs were harvested and reseeded to monitor PlGF secretion levels in the cell-conditioned medium. PlGF expression levels stayed significantly higher throughout the 7-day period with 107.3 ± 13 pg/ml compared to the empty plasmid-transfected cells (50.48 ± 5.2 pg/ml) after 7 days (Figure 3(h)). Additionally, hBM-MSCs continued to grow up to 7 days, achieving over 400% growth with empty plasmid transfection and over 600% growth with PlGF transfection (Figure 3(i)). Together, these results show that a functional plasmid, PlGF can be overexpressed in hBM-MSCs without lowering viability and growth.

## 4. Discussion

Due to the therapeutic potential of genetically modifying bone marrow-derived MSCs, there is a strong demand for an effective method to transiently transfect hBM-MSC. However, many methods face challenges such as low efficiency or low cell quality. Before conducting this study, we first tested the Lipofectamine 3000 system on hBM-MSCs due to reported success with Lipofectamine systems [19, 33]. However, we observed low cell recovery posttransfection (data not shown). That prompted us to compare Lipofectamine 3000 to 5 additional commercially available transfection systems from 3 different categories (lipid-, polymer-, and lipid-polymer-based systems) in order to find the best suited method for hBM-MSCs. Out of these transfection systems, Lipofectamine LTX [34], PEI [35], and Lipofectamine 3000 [36] had been previously reported to transfect DNA into hBM-MSCs whereas TransIT-2020, TransIT293, and jetPRIME had not been reported. TransIT-2020 was selected as the candidate system since it had the highest transfection efficiency without affecting cell recovery based on our multivariate screen with the GFP reporter plasmid. A high cell recovery is especially important for therapeutic purposes since clinically relevant doses of hBM-MSCs can require millions of cells [37, 38].

Previous studies showed that serum could affect transfection efficiency [39]; therefore, Opti-MEM was used as a serum-free medium to incubate the DNA and TransIT-2020 complex with the cells for 4 hours (4h-OM), similar to other serum-free incubation protocols [19], or 24 (24h-OM) hours. Using the 4h-OM condition, an appreciable range of GFP expression (26–35%), high cell viability (85–96%), and low cytotoxicity (1.7–2.3%) was observed among the 5 donors, highlighting the importance of testing multiple donors to verify method applicability. Since LDH is released when the plasma membrane is damaged, LDH data, along with viability data from Trypan blue and SYTOX Orange, provided a complementary mean of determining cell health after transfection in our study.

Although GFP is an excellent reporter gene for assessing and optimising transfection efficiency, it has little relevance to enhancing biological features that could be clinically useful. PlGF was chosen as a proof-of-concept plasmid due to its role in angiogenesis [40] and neuroprotection where hBM-MSCs overexpressing PlGF using an adenoviral vector improved cerebral ischemia in a rat model [27], known MSC functions. Transfection of hBM-MSCs with pCMV6-AC-PlGF using the optimised 4h-OM transfection workflow served as proof-of-concept, showing how a secreted protein, normally expressed at low levels in hBM-MSCs [41], can be effectively overexpressed for up to 7 days without significantly affecting cell integrity. Transfection led to an increase of PlGF production up to 220-fold when scaled up to a T75 which is significant since a therapeutic effect was seen *in vivo* when a 300-fold increase was obtained using an adenoviral vector at a multiplicity of infection of 3000 pfu/cell [27]. The successful scale up of the 4h-OM condition using the TransIT-2020 transfection method from a 96-well plate to a T75 is of great importance as it shows the potential of the scalability of the method which could be used to produce clinically relevant doses of genetically modified hBM-MSC. The significant increase in cell growth with PlGF-transfected cells compared to an empty vector could be related to PlGF's ability to increase proliferation in various cell types such as endothelial cells and fibroblasts [42].

With up to 26–35% transfection efficiency using a GFP reporter plasmid with multiple donors, our method has comparable transfection efficiency and viability to other lipid and polymer transfection methods [19, 22, 43]. Although efficiencies as high as 58% have been reported with transfection regents such as Lipofectamine 2000, results have been inconsistent since efficiencies lower than 10% have been reported for the same reagent [33, 44]. In addition, our study emphasized cell quality aspects through analysis of cytotoxicity and retention of MSC identity. hBM-MSCs were >95% positive for CD90, CD105, and CD73 surface markers after transfection which may not be the case with all reported transfection methods. Cells also showed lower cytotoxicity in all donors with TransIT-2020 compared to other transfection methods tested for LDH [44]. In addition, most other transfection studies have only used reporter plasmids and have not tested their workflow using a plasmid encoding for a gene of interest, such as PlGF. Furthermore, while viral transfection can generate higher transfection efficiencies approaching greater than 90% as seen in adenovirus transfection of BMP-2 in MSCs, limitations on transfection workflows as a result of increased observed immunogenicity prevent viral transduction from capitalizing higher MOIs required for higher transfection rates. As a result, lower MOIs that do not induce immunogenic effects share transfection efficiencies (20–30%) on par with the nonviral counterparts as shown in this study expressing the benefits of nonviral transfection [45]. In summary, we report an efficient and accessible means of transient hBM-MSC transfection which does not adversely affect cell viability, identity, and yield, important parameters of hBM-MSC quality for clinical use [11, 12]. When applied to hBM-MSCs, this method can serve to improve hBM-MSC cellular therapy by increasing the abundance of bioactive molecules such as PlGF that show potential for therapies focusing on regenerative medicine. This 4h-OM optimised means of transfection based on TransIT-2020 is a promising way to genetically modify hBM-MSCs but warrants further study to successfully translating it to the clinic. Studies such as indication-specific preclinical investigations as well as tests for tumourigenicity and immunogenicity would be required for clinical translation.

## Data Availability

The data used to support the findings of this study are available from the corresponding author upon request.

## Supplementary Materials

Supplementary 1 Supplementary Table 1: in-process data for working cell banks of hBM-MSCs. Supplementary Table 2: information on antibodies for hMSC surface marker staining. Supplementary Table 3: differentiation of transfected and untransfected hBM-MSCs into osteocytes, adipocytes, and chondrocytes.

Supplementary 2 Supplementary Figure 1: transfection of hBM-MSC #15 using TransIT-2020 does not affect cell viability and surface marker profiling. (a) Representative fluorescent overlay images of GFP+ cells (green) and cells (bright-field) for TransIT-2020-transfected hBM-MSCs after 24 hours at 3 different R/DNA ratios in 6-well plates. Scale bars represent 1000 *μ*m. (b) Representative flow profiles of transfected cells after a 24-hour transfection under all 3 R/DNA ratios using TransIT-2020, where reagent only (R) was used as a control. Unstained cells (US) and untransfected cells (UT) were also included as controls. (c) Percentage of GFP+ cells quantified by flow cytometry. (d) Mean fluorescence intensity (MFI) of transfected cells quantified by flow cytometry. (e) Percentage of viable cells quantified by flow cytometry using SYTOX Orange. (f) Quantification percentage CD90+, CD105+, and CD73+ cells 24 hour after transfection under all 3 R/DNA ratios using Trans-IT 2020, where reagent only (R) was used as a control. Results are from 3 independent experiments with technical duplicates. Error bars represent S.E.M. Statistical significance was obtained using a one-tailed *t*-test, ^∗^ *p* < 0.05. Supplementary Figure 2: diagram of the different incubation conditions to further optimise transfection using TransIT-2020 with a GFP reporter plasmid. Supplementary Figure 3: effect of Opti-MEM incubation on TransIT-2020 transfection of hBM-MSCs. (a) Representative fluorescence images of GFP-transfected cells using TransIT-2020 where green represents GFP+ cells. Transfection was done on hBM-MSC #37RB culture after 24 hours at 3 different transfection conditions. Scale bars represent 500 *μ*m. Following incubation for 15 minutes of R/DNA complexes, cells were kept either in complete medium for 24hours (24h-CM) or in Opti-MEM for 4 hours before being switched to CM for the remaining 20 hours (4h-OM) or in Opti-MEM for a total of 24 hours (24h-OM). (b) Quantification of percent GFP+ cells of 3 transfection conditions and their respective transfection reagent only (R) control. (c) Quantification of GFP mean fluorescence intensity (MFI) of transfected cells by flow cytometric analyses. (d) Percentage of viable cells quantified by flow cytometry using SYTOX Orange. Results are from 4 independent experiments with technical duplicates, and error bars represent S.E.M. Statistical significance was obtained using a one-tailed *t*-test for panel 1b and multiple *t*-tests followed by a Holm-Sidak correction for panel 1c (^∗∗^ *p* < 0.01, ^∗∗∗^ *p* < 0.001).
