# Supplementary material for: Efficient Nonviral Transfection of Human Bone Marrow Mesenchymal Stromal Cells Shown Using Placental Growth Factor Overexpression
Source: Stem Cells Int. 2018 Dec 24;2018:1310904. doi: 10.1155/2018/1310904 (PMC6323439; doi:10.1155/2018/1310904)
Supplement: Supplementary 1 — Supplementary Table 1: in-process data for working cell banks of hBM-MSCs. Supplementary Table 2: information on antibodies for hMSC surface marker staining. Supplementary Table 3: differentiation of transfected and untransfected hBM-MSCs into osteocytes, adipocytes, and chondrocytes. [file 1310904.f1.pdf]

Supplementary Table 1. In-process data for working cell banks of hBM-MSCs.

[a]**Donor** ID/gender/age	[b]**P1-P2 growth** MSCs plated per cm^2 / cells harvested per cm^2 / days	[c]**P1-P2** Population doublings	[b]**P2-P3 growth** MSCs plated per cm^2 / cells harvested per cm^2 / days	[c]**P2-P3** Population doublings	[d]**Differentiation** osteocytes / adipocytes / chondrocytes	[e]**Surface Marker Expression**
hBM-MSC#15/M/22	1571/9523/3	2.60	4286/23347/3	2.45	P3 testing + / + / +	>95% CD105, CD73 and CD90, <2% CD45, CD34
hBM-MSC#12RB/M/33	2720/22800/4	3.07	1429/44229/6	4.95	P1 testing + / + / +	>95% CD105, CD73 and CD90, <5% CD45, CD34
hBM-MSC#37RB/M/43	2857/28571/4	3.32	1429/18286/4	3.68	P1 testing + / + / +	>95% CD105, CD73 and CD90, <2% CD45, CD34
hBM-MSC#48RB/M/23	1429/15238/4	3.42	1429/21371/4	3.90	P1 testing + / + / +	>95% CD105, CD73 and CD90, <5% CD45, CD34
hBM-MSC#56RB/M/22	1429/20571/4	3.85	1429/15771/4	3.46	P1 testing + / + / +	>95% CD105, CD73 and CD90, <2% CD45, CD34
hBM-MSC#85RB/M/31-45	1429/24000/4	3.00	1429/27200/4	4.32	P1 testing + / + / +	>90% CD105, CD73 and CD90, <2% CD45, CD34

[a]Healthy donors were identified by anonymous identifier/gender/age. h=human, BM=bone marrow, #15 cells were grown in Mesencult SF medium and RB cells were grown in RB complete medium.

[b]P1-P2 or P2-P3 growth was defined as number of MSC cells plated per cm^2 / number of cells harvested per cm^2 at / days of incubation. Cells were harvested at 70-90% confluence.

[c]Cell population doublings were calculated as $n = \log_2$ (harvested viable cells / initial seeded viable cells)

[d]Differentiation was assayed by chemical staining after culture in inductive media (+ = present).

[e]Cell surface profiling by flow cytometry

Supplementary Table 2. Information on antibodies for hMSC surface marker staining.

Reagent	Company	Clone number	Catalog number	Application
CD34-PE	eBioscience	4H11	12-0349-42	Initial chacterization of hBM-MSC #15 surface markers[a]
CD45-FITC	eBioscience	HI30	11-0459-42	
CD73-PE-Cy7	eBioscience	AD2	25-0739-42	
CD90-PerCP-Cy5.5	eBioscience	5E10	45-0909-41	
CD105-APC	eBioscience	SN6	17-1057-41	
CD73-BUV737	BD Biosciences	AD2	565395	Chacterization of hBM-MSC surface markers after transfection[b]
CD90-APC	BD Biosciences	5E10	559869	
CD105-BUV395	BD Biosciences	266	563803	
BD Stemflow Human MSC Analysis Kit	BD Biosciences	See manufacture's specification sheet	562245	Chacterization of hBM-MSC #37RB surface markers[c]

[a]Cells and antibodies were incubated for 30 minutes, washed, then live cells were analysed with PBS 2% FCS.

[b]Antibody mixes were prepared in BD Horizon™ Brilliant Stain Buffer (BD biosciences, cat.# 659611, BD biosciences). Cells were stained with antibodies for 30 minutes, washed, then live cells were analysed with PBS 2% FCS.

[c]Followed procedure according to manufacture's protocol. Kit contains antibodies for CD73, CD90, CD105, CD44, CD34, CD45, CD11b, CD19, and HLA-DR

Supplementary Table 3. Differentiation of transfected and untransfected hBM-MSCs into osteocytes, adipocytes and chondrocytes.

**Donor ID**	[a]Differentiation of untransfected cells osteocytes / adipocytes / chondrocytes	[a]Differentiation after GFP transfection osteocytes / adipocytes / chondrocytes
hBM-MSC#12RB	+ / + / +	+ / + / +
hBM-MSC#37RB	+ / + / +	+ / + / +
hBM-MSC#48RB	[b]ND	[b]ND
hBM-MSC#56RB	+ / + / +	+ / + / +
hBM-MSC#85RB	+ / + / +	+ / + / +

[a]Differentiation was assayed by chemical staining after culture in inductive media (+ = present)
[b]Not determinded
