# Supplementary material for: Efficient Nonviral Transfection of Human Bone Marrow Mesenchymal Stromal Cells Shown Using Placental Growth Factor Overexpression
Source: Stem Cells Int. 2018 Dec 24;2018:1310904. doi: 10.1155/2018/1310904 (PMC6323439; doi:10.1155/2018/1310904)
Supplement: Supplementary 2 — Supplementary Figure 1: transfection of hBM-MSC #15 using TransIT-2020 does not affect cell viability and surface marker profiling. (a) Representative fluorescent overlay images of GFP+ cells (green) and cells (bright-field) for TransIT-2020-transfected hBM-MSCs after 24 hours at 3 different R/DNA ratios in 6-well plates. Scale bars represent 1000 μm. (b) Representative flow profiles of transfected cells after a 24-hour transfection under all 3 R/DNA ratios using TransIT-2020, where reagent only (R) was used as a control. Unstained cells (US) and untransfected cells (UT) were also included as controls. (c) Percentage of GFP+ cells quantified by flow cytometry. (d) Mean fluorescence intensity (MFI) of transfected cells quantified by flow cytometry. (e) Percentage of viable cells quantified by flow cytometry using SYTOX Orange. (f) Quantification percentage CD90+, CD105+, and CD73+ cells 24 hour after transfection under all 3 R/DNA ratios using Trans-IT 2020, where reagent only (R) was used as a control. Results are from 3 independent experiments with technical duplicates. Error bars represent S.E.M. Statistical significance was obtained using a one-tailed t-test, ∗ p < 0.05. Supplementary Figure 2: diagram of the different incubation conditions to further optimise transfection using TransIT-2020 with a GFP reporter plasmid. Supplementary Figure 3: effect of Opti-MEM incubation on TransIT-2020 transfection of hBM-MSCs. (a) Representative fluorescence images of GFP-transfected cells using TransIT-2020 where green represents GFP+ cells. Transfection was done on hBM-MSC #37RB culture after 24 hours at 3 different transfection conditions. Scale bars represent 500 μm. Following incubation for 15 minutes of R/DNA complexes, cells were kept either in complete medium for 24hours (24h-CM) or in Opti-MEM for 4 hours before being switched to CM for the remaining 20 hours (4h-OM) or in Opti-MEM for a total of 24 hours (24h-OM). (b) Quantification of percent GFP+ cells of 3 transf [file 1310904.f2.pdf]

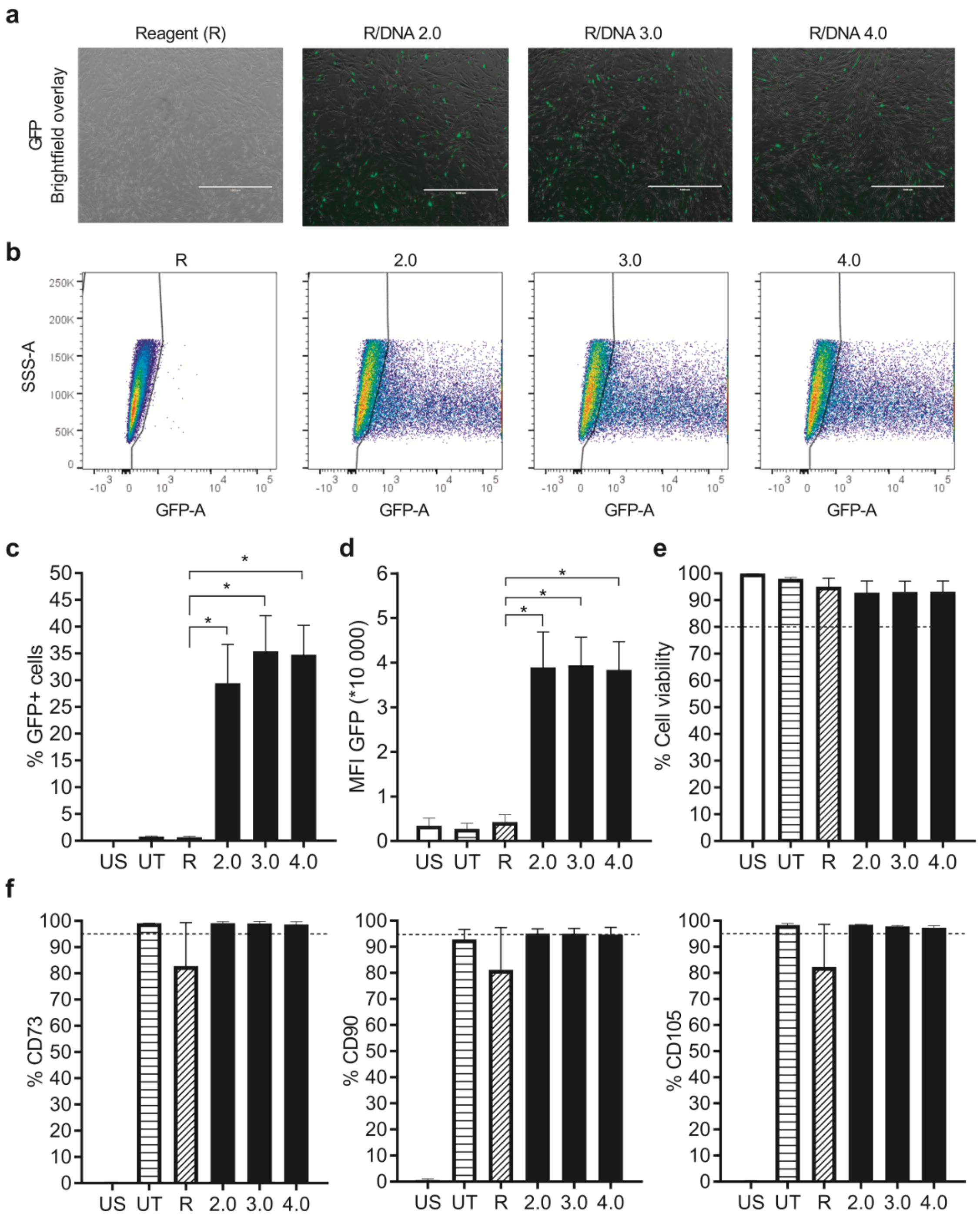

Supplementary Figure 1. Transfection of hBM-MSC #15 using TransIT-2020 does not affect cell viability and surface marker profiling. a) Representative fluorescent overlay images of GFP+ cells (green) and cells (bright-field) for TransIT-2020 transfected hBM-MSCs after 24

hours at 3 different R/DNA ratios in 6 well plates. Scale bars represent 1000 μm. b) Representative flow profiles of transfected cells after a 24 hour transfection under all 3 R/DNA ratios using TransIT-2020, where reagent only (R) was used as a control. Unstained cells (US) and untransfected cells (UT) were also included as controls. c) Percentage of GFP+ cells quantified by flow cytometry. d) Mean fluorescence intensity (MFI) of transfected cells quantified by flow cytometry. e) Percentage of viable cells quantified by flow cytometry using SYTOX Orange. f) Quantification percentage CD90+, CD105+ and CD73+ cells 24 hour after transfection under all 3 R/DNA ratios using Trans-IT 2020, where reagent only (R) was used as a control. Results are from 3 independent experiments with technical duplicates. Error bars represent S.E.M. Statistical significance was obtained using a one-tailed T test, *$p<0.05$.

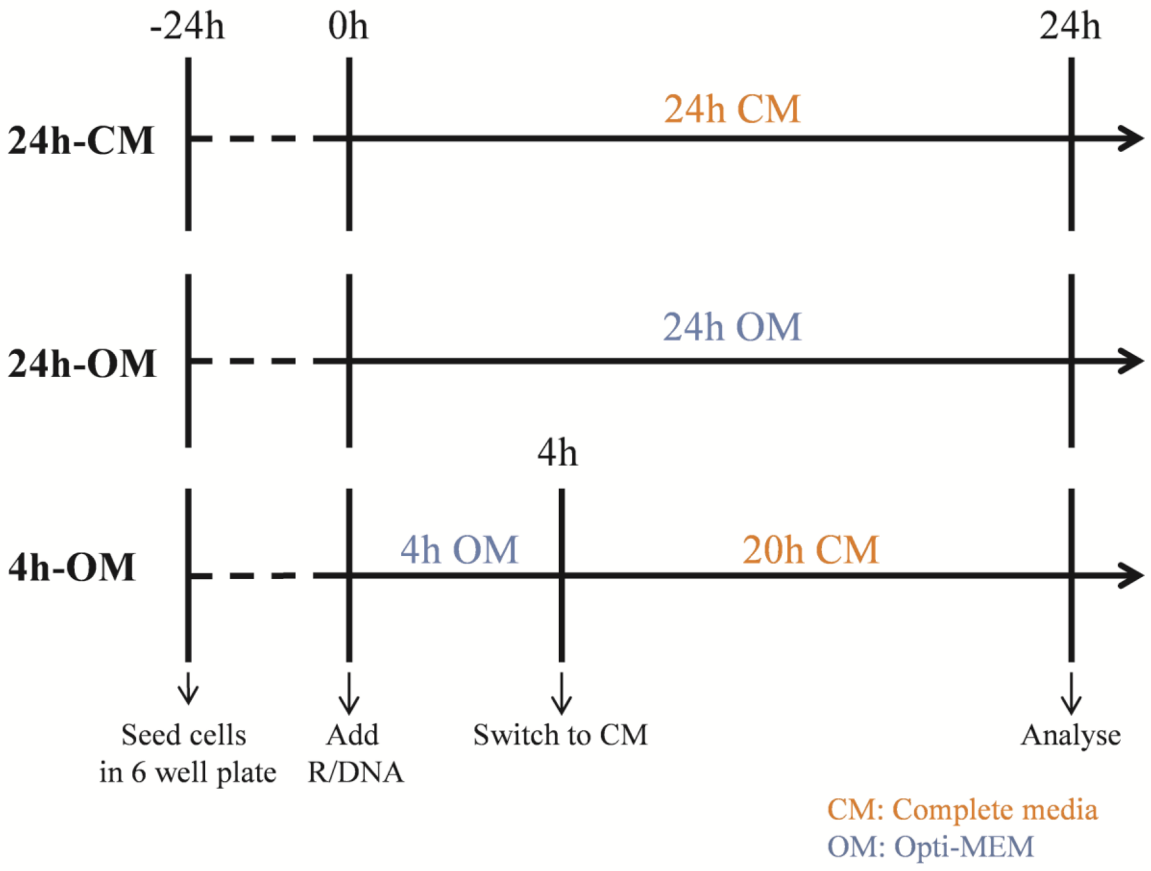

Supplementary Figure 2. Diagram of the different incubation conditions to further optimise transfection using TransIT-2020 with a GFP reporter plasmid.

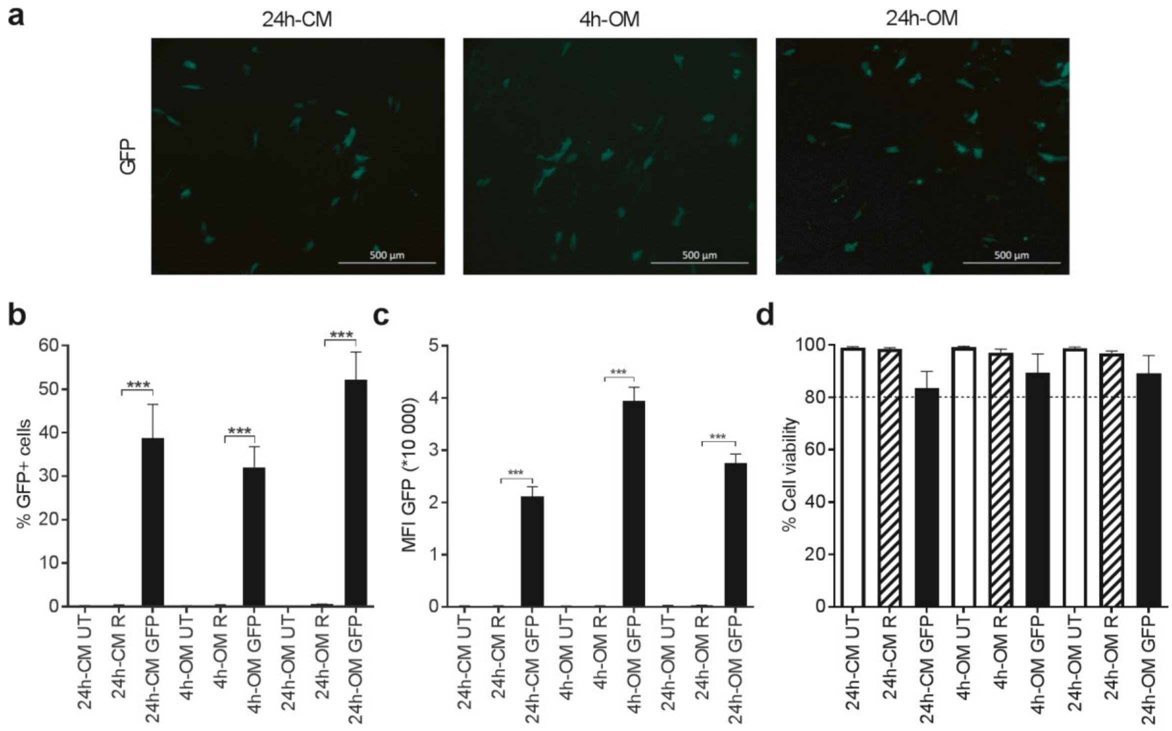

Supplementary Figure 3. Effect of Opti-MEM incubation on TransIT-2020 transfection of hBM-MSCs. a) Representative fluorescent images of GFP-transfected cells using TransIT-2020 where green represents GFP+ cells. Transfection was done on hBM-MSC #37RB culture after 24 hours at 3 different transfection conditions. Scale bars represent 500 µm. Following incubation for 15 minutes of R/DNA complexes, cells were either kept in complete medium for 24 hours (24h-CM), or in Opti-MEM for 4 hours before being switched to CM for the remaining 20 hours (4h-OM) or in Opti-MEM for a total of 24 hours (24h-OM). b) Quantification of percent GFP+ cells of 3 transfection conditions and their respective transfection reagent only (R) control. c) Quantification of GFP mean fluorescence intensity (MFI) of transfected cells by flow cytometric analyses. d) Percentage of viable cells quantified by flow cytometry using SYTOX Orange. Results are from 4 independent experiments with technical duplicates, and error bars represent

S.E.M. Statistical significance was obtained using a one-tailed T test for Panel 1b and a multiple T tests followed by a Holm-Sidak correction for Panel 1c, $**p<0.01$, $***p<0.001$.
